# Machine learning models on a web application to predict short-term postoperative outcomes following anterior cervical discectomy and fusion

**DOI:** 10.1186/s12891-024-07528-5

**Published:** 2024-05-21

**Authors:** Mert Karabacak, Abhiraj D. Bhimani, Alexander J. Schupper, Matthew T. Carr, Jeremy Steinberger, Konstantinos Margetis

**Affiliations:** Department of Neurosurgery, Mount Sinai Health System, 1468 Madison Ave, New York, NY 10029 USA

**Keywords:** Artificial intelligence, Machine learning, Outcome prediction, Web application, ACDF, Personalized medicine, Precision medicine, Spine surgery

## Abstract

**Background:**

The frequency of anterior cervical discectomy and fusion (ACDF) has increased up to 400% since 2011, underscoring the need to preoperatively anticipate adverse postoperative outcomes given the procedure’s expanding use. Our study aims to accomplish two goals: firstly, to develop a suite of explainable machine learning (ML) models capable of predicting adverse postoperative outcomes following ACDF surgery, and secondly, to embed these models in a user-friendly web application, demonstrating their potential utility.

**Methods:**

We utilized data from the National Surgical Quality Improvement Program database to identify patients who underwent ACDF surgery. The outcomes of interest were four short-term postoperative adverse outcomes: prolonged length of stay (LOS), non-home discharges, 30-day readmissions, and major complications. We utilized five ML algorithms - TabPFN, TabNET, XGBoost, LightGBM, and Random Forest - coupled with the Optuna optimization library for hyperparameter tuning. To bolster the interpretability of our models, we employed SHapley Additive exPlanations (SHAP) for evaluating predictor variables’ relative importance and used partial dependence plots to illustrate the impact of individual variables on the predictions generated by our top-performing models. We visualized model performance using receiver operating characteristic (ROC) curves and precision-recall curves (PRC). Quantitative metrics calculated were the area under the ROC curve (AUROC), balanced accuracy, weighted area under the PRC (AUPRC), weighted precision, and weighted recall. Models with the highest AUROC values were selected for inclusion in a web application.

**Results:**

The analysis included 57,760 patients for prolonged LOS [11.1% with prolonged LOS], 57,780 for non-home discharges [3.3% non-home discharges], 57,790 for 30-day readmissions [2.9% readmitted], and 57,800 for major complications [1.4% with major complications]. The top-performing models, which were the ones built with the Random Forest algorithm, yielded mean AUROCs of 0.776, 0.846, 0.775, and 0.747 for predicting prolonged LOS, non-home discharges, readmissions, and complications, respectively.

**Conclusions:**

Our study employs advanced ML methodologies to enhance the prediction of adverse postoperative outcomes following ACDF. We designed an accessible web application to integrate these models into clinical practice. Our findings affirm that ML tools serve as vital supplements in risk stratification, facilitating the prediction of diverse outcomes and enhancing patient counseling for ACDF.

**Supplementary Information:**

The online version contains supplementary material available at 10.1186/s12891-024-07528-5.

## Background

Anterior cervical discectomy and fusion (ACDF) is a common surgical procedure in the treatment of cervical spine conditions such as spondylosis or stenosis, causing symptoms such as radiculopathy and/or myelopathy [1, 2]. The anterior approach enables direct decompression of the spinal cord and reconstruction of the anterior column of the spine while providing access to the cervical spine along anatomic planes [3, 4]. According to the recent literature, the frequency of ACDF has increased by up to 400% since 2011 [5]. The increased practice of ACDF underscores the need to anticipate adverse postoperative outcomes preoperatively [6–10].

In an effort to control healthcare costs, emphasis is being placed on the use of registries and databases to track and establish risk-adjusted estimates for these outcomes. This has necessitated clinicians to manage extensive volumes of complex data, sparking the need for robust analytical techniques [11]. Machine learning (ML) algorithms, capable of leveraging high-dimensional clinical data, are increasingly employed to develop accurate patient risk assessment models, contribute to the development of guidelines, and adjust care to individual patient needs, thereby influencing healthcare decisions. These algorithms present several advantages over traditional prognostic models, often employing some form of linear or logistic regression. Firstly, ML seldom requires prior knowledge of primary predictors [12]. Secondly, these advanced ML algorithms often impose fewer constraints on the number of predictors used for a given dataset than logistic regression, proving beneficial in handling large datasets with numerous predictors, where associations between predictors and outcomes are not always obvious. Lastly, these algorithms can identify complex, nonlinear relationships within datasets, which are often overlooked by regression-based models [13]. Owing to these advantages, ML algorithms frequently outperform regression methods in terms of reliability and accuracy when applied to identical datasets [14, 15].

Several studies have demonstrated the predictive potential of ML models for various spinal procedures and pathologies, including ACDF [11, 16–25]. Yet, a vast majority of these investigations predominantly exist as feasibility studies, with a limited contribution towards the practical application of these models potentially in clinical environments. Our study seeks to address this gap by developing ML models focused on the prediction of short-term adverse postoperative outcomes after ACDF for degenerative cervical disease. We focus on short-term outcomes because they have critical implications for hospital reimbursements, surgeon evaluations, and patient recovery and satisfaction. Following model development, we intend to incorporate these models into an accessible web application, thereby demonstrating their pragmatic value.

## Methods

**Fig. 1:**
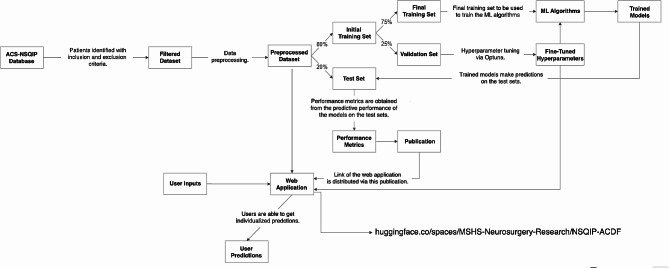
Methodology flowchart

The methodology employed is summarized with a flowchart in Fig. 1.

### Data source

Data for this study is from the American College of Surgeons (ACS) National Surgical Quality Improvement Program (NSQIP) database, which was queried to identify patients who underwent ACDF from 2014 to 2020. Detailed information about the database and data collection methods have been provided elsewhere [26].

### Guidelines

We followed Transparent Reporting of Multivariable Prediction Models for Individual Prognosis or Diagnosis (TRIPOD) [27] and Journal of Medical Internet Research (JMIR) Guidelines for Developing and Reporting Machine Learning Predictive Models in Biomedical Research [28].

### Study population

We queried the NSQIP database to identify patients in whom the following inclusion criteria were met: (1) Current Procedural Terminology (CPT) codes for ACDF surgery (22,551, 22,552, 22,554, and 22,585), (2) elective surgery, (3) operation under general anesthesia, and (4) surgical subspecialty neurosurgery or orthopedics. We excluded patients with the following criteria: (1) emergency surgery, (2) patients with any unclean wounds (defined by wound classes 2 to 4), (3) patients with sepsis, shock, or systemic inflammatory response syndrome 48 h before surgery, (4) patients with American Society of Anesthesiologists (ASA) physical status classification score of 4, 5 or not assigned, (5) patients still in hospital after 30 days since the NSQIP database captures postoperative outcomes up to 30 days after surgery, and (6) patients who were discharged to hospice, those who left against medical advice, and those who passed away. We excluded patients with 30-day mortality since our preliminary analysis of our patient cohort yielded only 19 patients with 30-day mortality, thus we could not investigate mortality as an outcome of interest due to the very few number of patients. Additionally, we excluded patients who underwent concomitant posterior cervical spinal surgery and total disc arthroplasty with relevant CPT codes (22,590, 22,595, 22,600, 22,614, 22,856, 22,858, 22,861, and 22,864). We reviewed the International Classification of Diseases (ICD) 10 codes assigned to the patients as principal diagnoses to further identify those undergoing surgery for degenerative diseases. Using ICD codes, patients with diagnoses of a fracture, neoplasm, or infection were also excluded. To avoid the effect of any confounding effect from rare pathologies and ICD-10 coding errors, we excluded cases with ICD codes that were utilized less than 50 times in the total patient population.

### Predictor variables and outcomes of interest

Variables from the NSQIP database that were supposed to have been known preoperatively were included as predictor variables. These included (1) demographic information such as age, sex, race, Hispanic ethnicity, height, weight, transfer status; (2) comorbidities and disease burden such as current smoker within one year, diabetes mellitus requiring therapy, dyspnea, ventilator dependency, history of severe chronic obstructive pulmonary disease (COPD), ascites within 30 days prior to surgery, congestive heart failure within 30 days prior to surgery, hypertension requiring medication, acute renal failure, currently requiring or on dialysis, disseminated cancer, presence of open wounds, steroid or immunosuppressant for a chronic condition, malnourishment, bleeding disorders, preoperative transfusion of ≥ 1 unit of whole/packed RBCs within 72 h prior to surgery, the ASA classification, functional status prior to surgery; (3) preoperative laboratory values such as serum sodium, blood urea nitrogen (BUN), serum creatinine, serum albumin, total bilirubin, serum glutamic-oxaloacetic transaminase (SGOT), alkaline phosphatase, white blood cell (WBC) count, hematocrit, platelet count, partial thromboplastin time (PTT), International Normalized Ratio of prothrombin time (PT) values, PT; (4) operative variables such as surgical specialty, single- versus multiple-level surgery.

The outcomes under investigation included prolonged LOS, non-home discharges, 30-day readmissions, and major complications. We defined the prolonged LOS as total LOS exceeding 90% of the entire patient population, which equated to ≥ 3 days. The discharge destination variable was dichotomized to delineate non-home discharges. In instances where patients necessitated further levels of care post-discharge, a non-home discharge destination was classified. This category incorporated destinations such as ‘Rehab’, ‘Skilled Care, Not Home’, ‘Separate Acute Care’, ‘Unskilled Facility Not Home’, and ‘Multi-level Senior Community’. Discharges to a ‘Facility Which Was Home’ were categorized as home discharges in addition to discharges to ‘Home’. Patients considered to have experienced major complications if they developed one or more of the following after surgery: deep incisional or organ/space surgical site infections, wound dehiscence, reintubation, pulmonary embolism, prolonged mechanical ventilation beyond 48 h, renal dysfunction or outright failure necessitating dialysis, cardiac arrest, myocardial infarction, hemorrhage requiring transfusion, deep venous thrombosis, sepsis, or septic shock. The NSQIP database also contained data on some less serious postoperative complications like superficial surgical site infection, pneumonia, and urinary tract infection, but these were not classified as major for the purposes of this analysis. Any patients with missing data for any of the four primary outcome measures being examined were omitted from the related analytical procedures.

### Data preprocessing and partition

We employed imputation to avoid any bias that might arise by excluding patients with missing data. The k-nearest neighbor imputation algorithm was utilized to fill in missing values in continuous variables after discarding variables that had more than 25% missing data [29]. For categorical variables, missing values were filled in with ‘Unknown’ or ‘Unknown/Other’.

To provide adequate data for the phases of model development, validation, and testing, we divided the 2014 to 2020 data into three subsets in a 60:20:20 ratio for training, validation, and test sets, respectively. The training set was used for training the ML models, the validation set for fine-tuning hyperparameters and calibration, and the test set for evaluating the models’ performance.

To address potential class imbalance in the training data, we employed the Synthetic Minority Over-sampling Technique (SMOTE) prior to model training. SMOTE counteracts skewed class distributions by artificially generating new examples belonging to the minority class, rather than duplicating existing samples [30]. This approach grows the number of instances from the under-represented class and has been shown to improve model performance compared to simply replicating minority samples. Applying SMOTE ensured adequate representation of all classes and avoided learning bias towards majority groups during the training process.

### Model development and performance evaluation

We built our prediction models using five different ML algorithms. These ML algorithms comprised a transformer-based algorithm named TabPFN [31], a neural network-based approach called TabNET [32], two gradient boosting algorithms, specifically XGBoost [33] and LightGBM [34], and a decision-tree-based algorithm Random Forest [35]. In order to maximize these models’ discriminatory abilities, we utilized the Optuna optimization library [36], employing the area under the receiver operating characteristic (AUROC) as the optimization standard. We used the Tree-Structured Parzen Estimator Sampler (TPESampler), a Bayesian optimization algorithm, to provide AUROC estimates that would guide the optimization process. The finalized prediction models were developed using the training sets and the hyperparameters optimized with Optuna. These optimized hyperparameters can be found in Supplementary Table 3. We applied Platt scaling, also recognized as isotonic regression, for model calibration [37]. All these analyses were performed on Python version 3.7.15 on the Google Colab platform.

We conducted a thorough evaluation of our models’ performance, both visually and numerically. The visual assessment was accomplished through the receiver operating characteristic (ROC) and precision-recall curve (PRC), while numerical metrics used for classification performance evaluation included AUROC, balanced accuracy, weighted area under PRC (AUPRC), weighted precision, and weighted recall. Calibration was evaluated using the Brier score.

We chose models for web application deployment based on their AUROC values. AUROC, a widely used performance metric in ML models, is particularly beneficial in binary classification tasks [38]. This measure assesses a model’s ability to differentiate between positive and negative samples across various classification thresholds. We chose AUROC as a primary measure due to its multiple advantages. First, it is not affected by class imbalance, making it a suitable choice for datasets with uneven class distribution. Second, it considers the complete range of classification thresholds, providing a thorough evaluation of model performance across diverse points. Third, AUROC quantifies the model’s ability to correctly rank instances irrespective of the chosen classification threshold. By distilling the model’s performance into a single value, AUROC simplifies the comparison process among different models or algorithms. As a result, it offers a reliable reflection of the model’s discriminative power and is thus an appropriate metric for model evaluation and selection across various applications.

To enhance our models’ interpretability, we used SHapley Additive exPlanations (SHAP) to determine the relative importance of predictor variables [39]. In addition, we used partial dependency plots (PDPs) to display the effect of individual variables on the predictions of the top-performing models.

### Web application

We developed a web application to allow users to make individual patient predictions. The top-performing models for each outcome were incorporated into this application. The source code for implementing these models online can be found on the Hugging Face platform, which a community-friendly site for sharing ML models. We have also included Supplementary Video 1 to demonstrate the web application’s functionality. The web application can be accessed via this link: https://huggingface.co/spaces/MSHS-Neurosurgery-Research/NSQIP-ACDF.

### Descriptive statistics

For continuous variables with a normal distribution, we reported means (± standard deviations), and for those with a non-normal distribution, we presented medians (interquartile ranges). The patient count was represented as percentages for categorical variables.

## Results

**Fig. 2:**
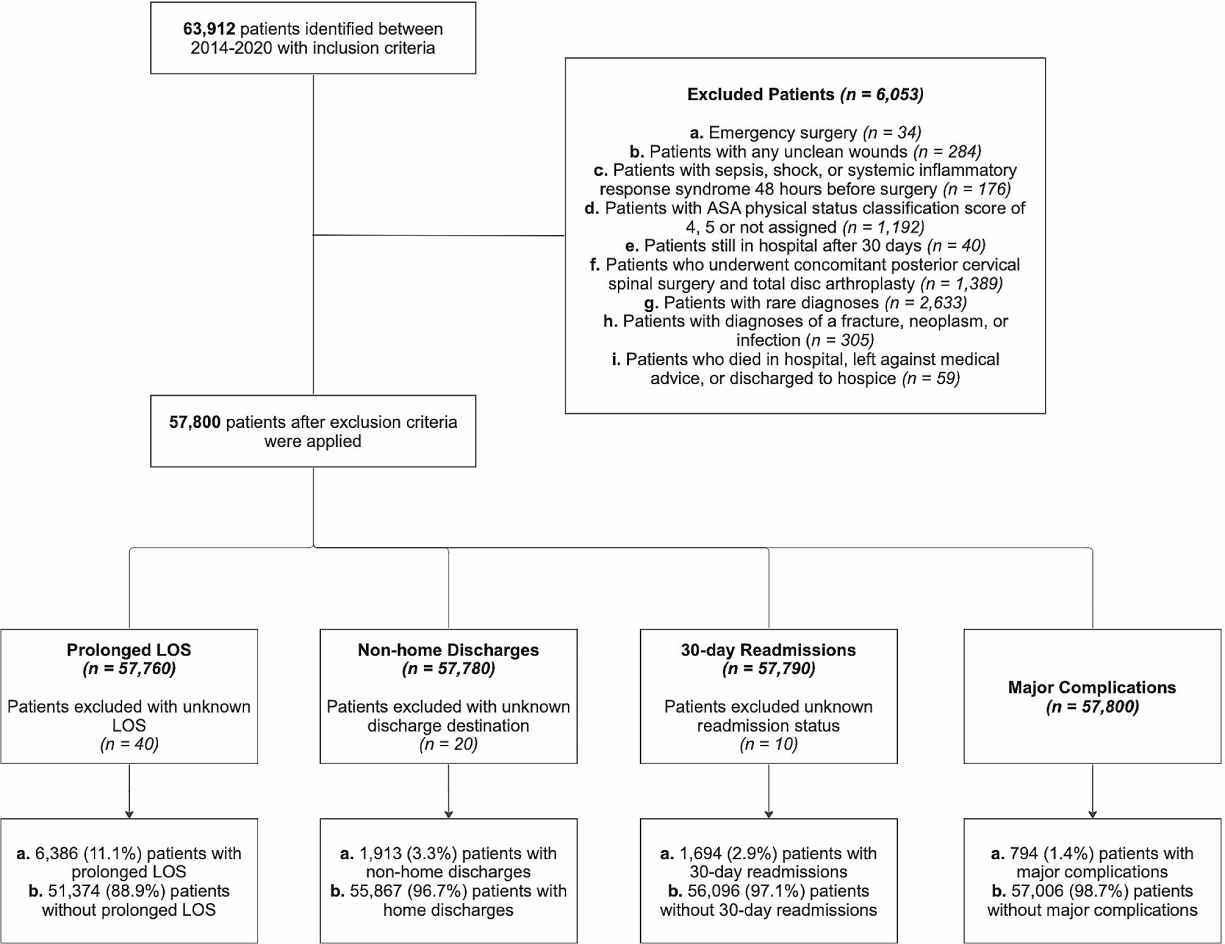
Patient selection flowchart

63,912 patients were identified with the inclusion criteria. Exclusion criteria were applied sequentially, and 6,053 patients were excluded (Fig. 2). After outcome-specific exclusion criteria were applied, there were 57,760 patients included in the analysis for the outcome prolonged LOS [*n* = 6,386 (11.1%) with prolonged LOS], 57,780 for the outcome non-home discharges [*n* = 1,913 (3.3%) with non-home discharges], 57,790 for the outcome 30-day readmissions [*n* = 1,694 (2.9%) with 30-day readmissions], and 57,800 for the outcome major complications [*n* = 794 (1.4%) with major complications. Characteristics of the patient population (*n* = 57,859) before the outcome-specific exclusion criteria were applied are presented in Table 1.

**Table 1:** Patient characteristics

Variables		Mean (± SD), Median (IQR), or *n* (%)
Age		56.0 (16.0)
Sex	Female	29,065 (50.2%)
	Male	28,794 (49.8%)
Race	White	45,483 (78.6%)
	Black or African American	6059 (10.5%)
	Asian	997 (1.7%)
	American Indian or Alaska Native	302 (0.5%)
	Native Hawaiian or Pacific Islander	160 (0.3%)
	Other/Unknown	4858 (8.4%)
Hispanic Ethnicity	No	50,365 (87.0%)
	Yes	3226 (5.6%)
	Unknown	4268 (7.4%)
Height (cm)		168 (± 15.2)
Weight (kg)		86.18 (± 27.22)
Transfer Status	Not transferred	57,501 (99.4%)
	Transferred	348 (0.6%)
	Unknown	10 (< 0.1%)
Current Smoker Status	No	43,719 (75.6%)
	Yes	14,140 (24.4%)
Diabetes Mellitus Requiring Therapy	No	48,005 (83.0%)
	Yes	9854 (17.0%)
Dyspnea	No	55,196 (95.4%)
	Yes	2663 (4.6%)
Functional Status	Independent	56,903 (98.4%)
	Partially Dependent	693 (1.2%)
	Unknown	214 (0.4%)
	Totally Dependent	49 (0.1%)
Ventilator Dependency	No	57,857 (> 99.9%)
	Yes	2 (< 0.1%)
History of Severe COPD	No	55,309 (95.6%)
	Yes	2550 (4.4%)
Ascites within 30 Days Prior to Surgery	No	57,856 (> 99.9%)
	Yes	3 (< 0.1%)
CHF within 30 Days Prior to Surgery	No	57,750 (99.8%)
	Yes	109 (0.2%)
Hypertension Requiring Medication	No	30,710 (53.1%)
	Yes	27,149 (46.9%)
Acute Renal Failure	No	57,841 (> 99.9%)
	Yes	18 (< 0.1%)
Currently Requiring or on Dialysis	No	57,798 (99.9%)
	Yes	61 (0.1%)
Disseminated Cancer	No	57,802 (99.9%)
	Yes	57 (0.1%)
Open Wound	No	57,780 (99.9%)
	Yes	79 (0.1%)
Steroid or Immunosuppressant for a Chronic Condition	No	55,876 (96.6%)
	Yes	1983 (3.4%)
Malnourishment	No	57,783 (99.9%)
	Yes	76 (0.1%)
Bleeding Disorder	No	57,272 (99.0%)
	Yes	587 (1.0%)
RBC Transfusion within 72 h Prior to Surgery	No	57,851 (> 99.9%)
	Yes	8 (< 0.1%)
Preoperative Serum Sodium		140.0 (± 3.0)
Preoperative Serum BUN		15.0 (± 6.0)
Preoperative Serum Creatinine		0.87 (± 0.25)
Preoperative WBC Count (x1000)		7.1 (± 2.6)
Preoperative Hematocrit		42.0 (± 5.0)
Preoperative Platelet Count (x1000)		247.0 (± 78.0)
Surgical Specialty	Neurosurgery	41,894 (72.4%)
	Orthopedics	15,965 (27.6%)
ASA Classification	1-No Disturb	1674 (2.9%)
	2-Mild Disturb	29,641 (51.2%)
	3-Severe Disturb	26,544 (45.9%)

**Fig. 3:**
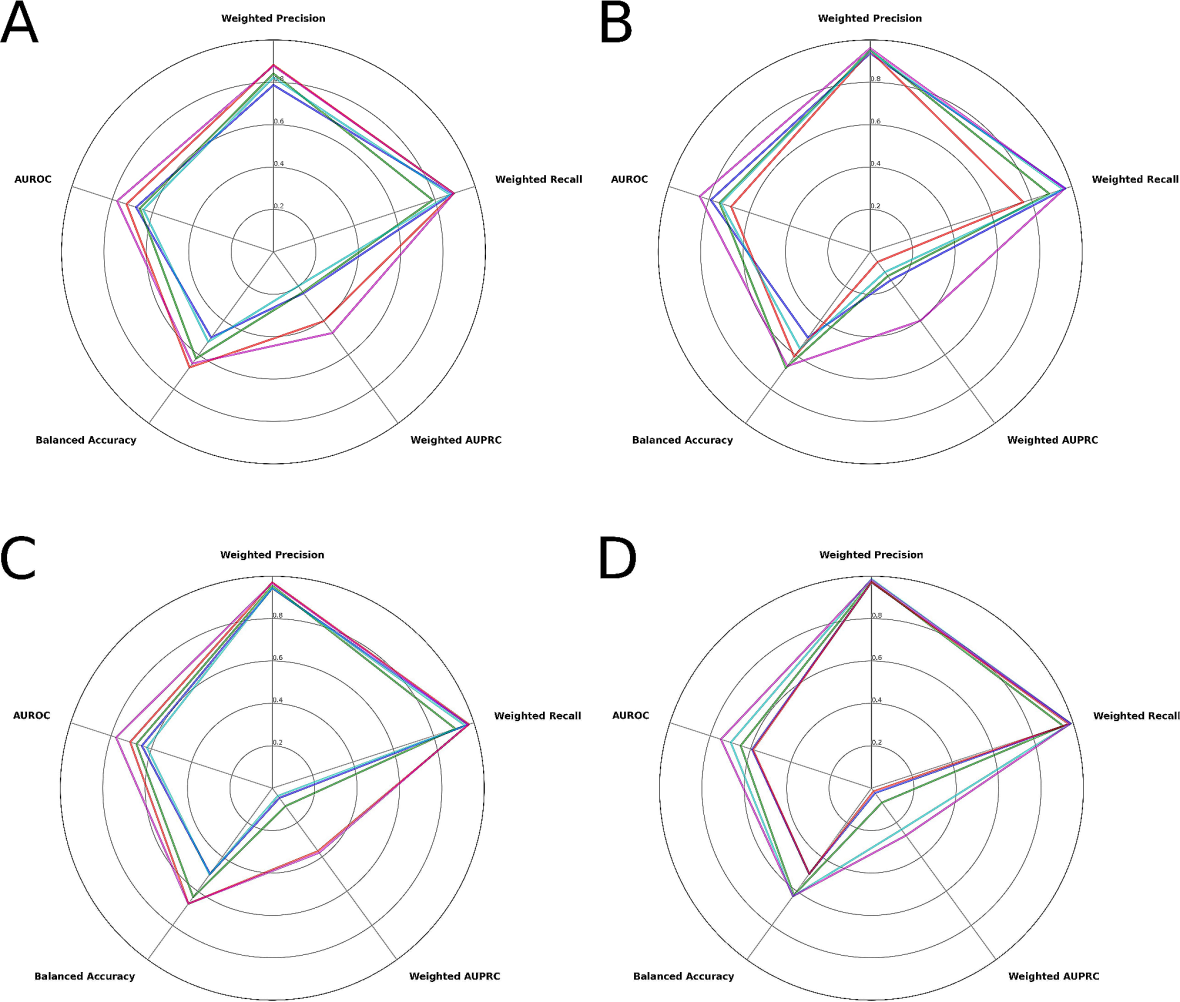
Algorithms’ radar plots for the outcomes (**A**) prolonged length of stay, (**B**) non-home discharges, (**C**) 30-day readmissions, and (**D**) major complications

Performance evaluation indicated that the top-performing models for each outcome were the models built with the Random Forest algorithm. The Random Forest models yielded AUROCs of 0.776 [95% confidence interval (CI), 0.766–0.792], 0.846 (95% CI, 0.809–0.855), 0.775 (95% CI 0.731–0.791), and 0.747 (0.702–0.779) in predicting prolonged LOS, non-home discharges, 30-day readmissions, and major complications respectively. These results indicate good success in distinguishing patients who had non-home discharges from those who did not. Fair discriminatory ability was seen in differentiatiating patients who experienced prolonged LOS, 30-day readmissions, and major complications [40]. Detailed information on these performance metrics is displayed in Table 2. Illustrated in Fig. 3 are radar plots, each corresponding to one of the four outcomes of interest. These charts serve as an instrument for multidimensional visualization, with each of the five axes standing for a separate performance indicator. The placement on each respective axis signifies the model’s performance in relation to that particular indicator. Consequently, these radar charts enable a comparative analysis of model performance across various metrics.

**Table 2:** Performance metrics of the models

	Algorithm	Weighted Precision (95% CI)	Weighted Recall (95% CI)	Weighted AUPRC (95% CI)	Balanced Accuracy (95% CI)	AUROC (95% CI)	Brier Score (95% CI)
Prolonged LOS	TabPFN	0.787 (0.78–0.794)	0.887 (0.881–0.893)	0.241 (0.233–0.249)	0.5 (0.491–0.509)	0.682 (0.672–0.702)	0.095 (0.09–0.1)
	TabNet	0.841 (0.834–0.848)	0.79 (0.783–0.797)	0.235 (0.227–0.243)	0.622 (0.613–0.631)	0.663 (0.64–0.672)	0.095 (0.09–0.1)
	XGBoost	0.882 (0.876–0.888)	0.894 (0.888–0.9)	0.404 (0.395–0.413)	0.674 (0.665–0.683)	0.729 (0.711–0.747)	0.081 (0.076–0.086)
	LightGBM	0.823 (0.816–0.83)	0.875 (0.869–0.881)	0.199 (0.192–0.206)	0.524 (0.515–0.533)	0.646 (0.635–0.665)	0.097 (0.092–0.102)
	Random Forest	0.878 (0.872–0.884)	0.894 (0.888–0.9)	0.473 (0.464–0.482)	0.651 (0.642–0.66)	0.776 (0.766–0.792)	0.078 (0.073–0.083)
Non-home Discharges	TabPFN	0.937 (0.933–0.941)	0.968 (0.965–0.971)	0.165 (0.158–0.172)	0.5 (0.491–0.509)	0.791 (0.755–0.805)	0.03 (0.027–0.033)
	TabNet	0.953 (0.949–0.957)	0.888 (0.882–0.894)	0.141 (0.135–0.147)	0.677 (0.668–0.686)	0.747 (0.694–0.751)	0.029 (0.026–0.032)
	XGBoost	0.948 (0.944–0.952)	0.759 (0.751–0.767)	0.059 (0.055–0.063)	0.611 (0.602–0.62)	0.69 (0.641–0.695)	0.031 (0.028–0.034)
	LightGBM	0.948 (0.944–0.952)	0.956 (0.952–0.96)	0.118 (0.112–0.124)	0.564 (0.555–0.573)	0.74 (0.73–0.777)	0.031 (0.028–0.034)
	Random Forest	0.961 (0.957–0.965)	0.964 (0.961–0.967)	0.402 (0.393–0.411)	0.666 (0.657–0.675)	0.846 (0.809–0.855)	0.024 (0.021–0.027)
30-day Readmissions	TabPFN	0.944 (0.94–0.948)	0.972 (0.969–0.975)	0.057 (0.053–0.061)	0.5 (0.491–0.509)	0.647 (0.626–0.685)	0.028 (0.025–0.031)
	TabNet	0.955 (0.951–0.959)	0.908 (0.903–0.913)	0.105 (0.099–0.111)	0.635 (0.626–0.644)	0.674 (0.648–0.712)	0.027 (0.024–0.03)
	XGBoost	0.971 (0.968–0.974)	0.976 (0.973–0.979)	0.367 (0.358–0.376)	0.673 (0.664–0.682)	0.705 (0.683–0.749)	0.02 (0.017–0.023)
	LightGBM	0.945 (0.941–0.949)	0.957 (0.953–0.961)	0.045 (0.041–0.049)	0.504 (0.495–0.513)	0.622 (0.595–0.655)	0.029 (0.026–0.032)
	Random Forest	0.968 (0.965–0.971)	0.971 (0.968–0.974)	0.376 (0.367–0.385)	0.674 (0.665–0.683)	0.775 (0.731–0.791)	0.022 (0.019–0.025)
Major Complications	TabPFN	0.972 (0.969–0.975)	0.986 (0.984–0.988)	0.028 (0.025–0.031)	0.5 (0.491–0.509)	0.592 (0.585–0.672)	0.014 (0.012–0.016)
	TabNet	0.977 (0.974–0.98)	0.951 (0.947–0.955)	0.084 (0.079–0.089)	0.623 (0.614–0.632)	0.649 (0.602–0.699)	0.014 (0.012–0.016)
	XGBoost	0.972 (0.969–0.975)	0.979 (0.976–0.982)	0.018 (0.016–0.02)	0.499 (0.49–0.508)	0.587 (0.526–0.612)	0.014 (0.012–0.016)
	LightGBM	0.987 (0.985–0.989)	0.989 (0.987–0.991)	0.241 (0.233–0.249)	0.63 (0.621–0.639)	0.698 (0.617–0.717)	0.011 (0.009–0.013)
	Random Forest	0.983 (0.981–0.985)	0.987 (0.985–0.989)	0.276 (0.268–0.284)	0.629 (0.62–0.638)	0.747 (0.702–0.779)	0.012 (0.01–0.014)

**Fig. 4:**
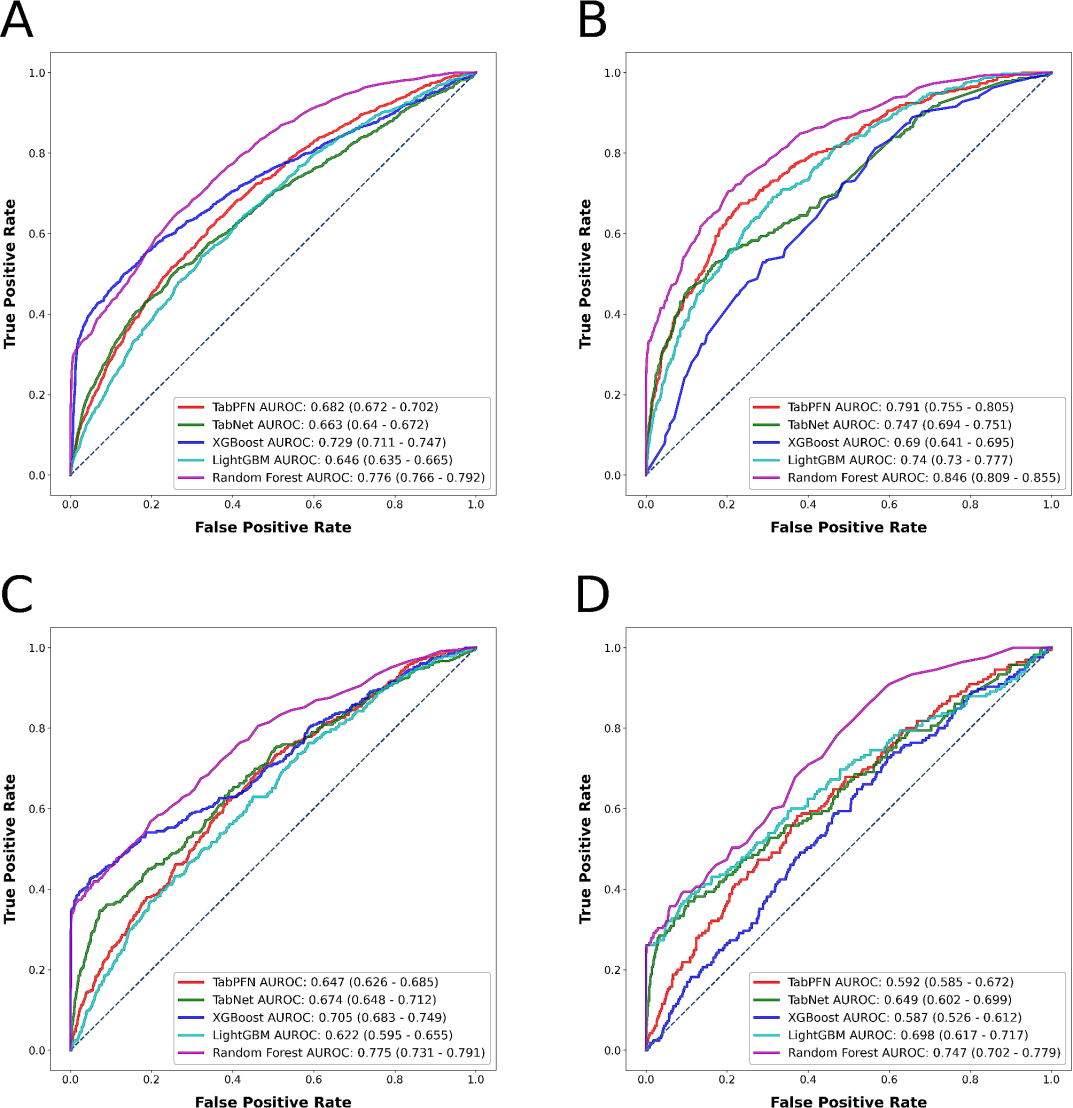
Algorithms’ receiver operating characteristics for the outcomes (**A**) prolonged length of stay, (**B**) non-home discharges, (**C**) 30-day readmissions, and (**D**) major complications

**Fig. 5:**
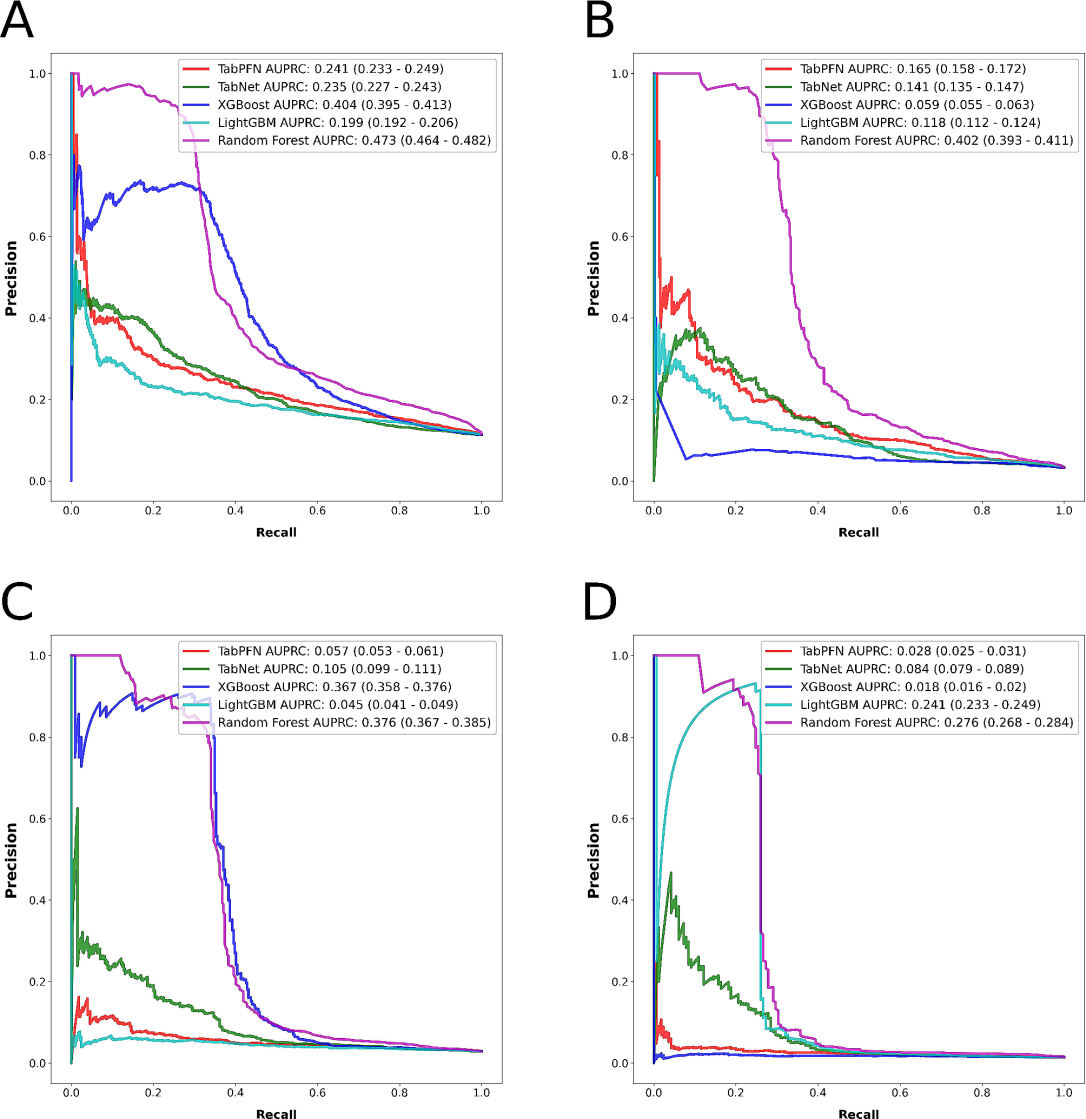
Algorithms’ precision-recall curves for the outcome (**A**) prolonged length of stay, (**B**) non-home discharges, (**C**) 30-day readmissions, and (**D**) major complications

**Fig. 6:**
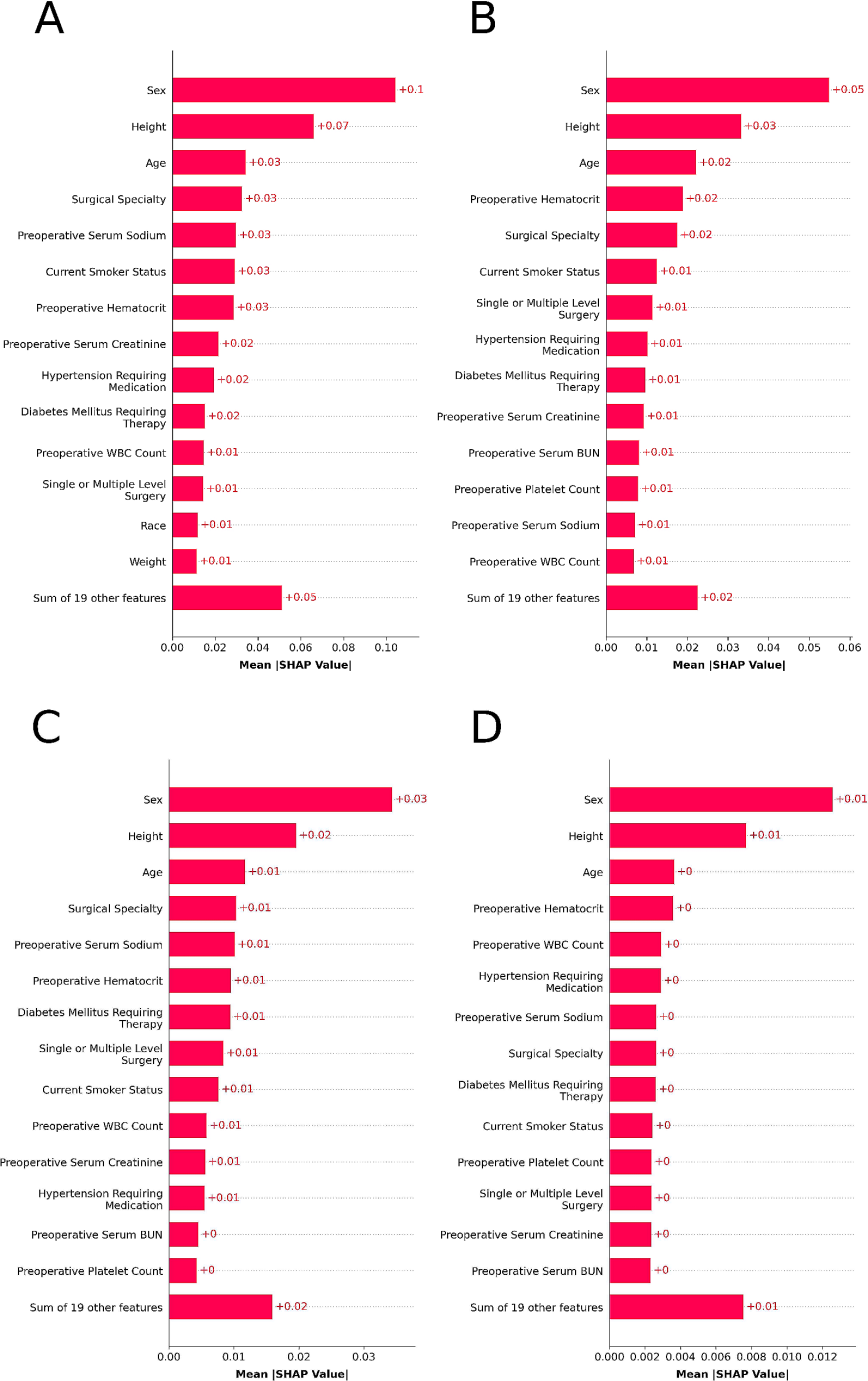
The 15 most important features and their mean SHAP values for the model predicting the outcome (**A**) prolonged length of stay with the Random Forest algorithm, (**B**) non-home discharges with the Random Forest algorithm, (**C**) 30-day readmissions with the Random Forest algorithm, and (**D**) major complications with the Random Forest algorithm

Figures 4 and 5, respectively, illustrate the ROCs and PRCs for the three outcomes, while Fig. 6 presents the SHAP bar plots for each outcome’s top-performing model. SHAP bar plots for other algorithms for each outcome are available in Supplementary Fig. 1 through 4. SHAP bar plots give a general overview of the significance of features in a model. Each bar in these plots represents the importance of a feature, with its length corresponding to the average absolute SHAP value across all instances. This measure of importance shows the average effect a feature has on the model’s prediction. The features are arranged according to their significance, with the most influential at the top.

Moreover, to better understand how individual feature values influence the model’s predictions, we refer to Supplementary Figs. 5–8, which present the PDPs for the models built with the Random Forest algorithm, each for one of the four outcomes of interest. As an illustration, Figure Supplementary Fig. 5 displays a non-linear curve for ‘Age’, indicative of a non-linear association between the feature ‘Age’ and the outcome, prolonged LOS. This underscores the advantage of ML algorithms in capturing non-linear relationships between variables and outcomes, a strength that traditional regression algorithms may not possess.

## Discussion

The goal of our study was to develop ML models capable of predicting short-term adverse postoperative outcomes following ACDF. Furthermore, to make our models more accessible, we developed a web application that allows healthcare professionals to input patient data and receive predicted risks for each outcome. This web application has the potential to serve as a valuable tool for clinicians by facilitating the estimation of a patient’s risk of adverse outcomes following ACDF. These models can aid clinicians in identifying patients at high risk of adverse outcomes following ACDF, thus enabling more informed patient counseling prior to the procedure.

When interpreting the metrics used to assess model performance, it is crucial to handle with care and understand the use of imbalanced datasets for ML classification tasks. We used metrics such as balanced accuracy, weighted precision, weighted recall, and weighted AUPRC to evaluate our models’ classification performance. These metrics consider the data’s class distribution, assigning more importance to the minority class [41–43]. This facilitates a just evaluation of the model’s performance across both classes and a broader perspective of the model’s effectiveness, taking into account the class distribution in the data. Conversely, the unweighted versions of these metrics might not be reliable in situations with imbalanced datasets as they overlook the class distribution and could present a misleading impression of good performance by neglecting the minority class. Furthermore, interpreting AUPRC can be more complex than another area under the curve metric, AUROC, due to its distinctive baselines. AUROC employs a baseline of 0.5, depicting a random classifier’s performance, whereas the baseline for AUPRC is the proportion of positive examples in the dataset [44]. This can result in significantly lower AUPRC values than AUROC, especially for datasets with a small fraction of positive examples, like many real-world medical datasets. Nevertheless, AUPRC might be more relevant for a particular problem, but it is often reported less frequently than AUROC due to its lower absolute values. For instance, in our study, the weighted AUPRC for the Random Forest model predicting prolonged LOS was 0.473 (95% CI, 0.464–0.482), while the prolonged LOS rate was 0.112, representing the baseline. Lastly, we evaluated the models’ calibration using the Brier score, a measure of the average squared disparity between predicted and actual probabilities [45, 46]. A model calibrated well will have a Brier score close to zero, implying that the predicted probabilities align closely with the actual probabilities.

One interesting finding was that Random Forest models outperformed other modern algorithms like XGBoost and LightGBM in terms of predictive performance across all outcomes. Despite being around for many years [35], tree-based ensamble methods like Random Forest remain robust and powerful approaches for prediction problems. The Random Forest algorithm creates numerous randomized decision trees and aggregates their predictions, allowing it to capture complex nonlinear relationships and high-order interactions between variables [47]. In addition, their ensemble nature makes them resistant to overfitting [48]. In contrast, more recent boosting methods like XGBoost [33] and LightGBM [34] also build ensembles of trees, but do so sequentially, focusing on misclassified examples in each iteration. While this can improve predictive accuracy, it may also increase overfitting risk compared to the Random Forest algorithm [49]. The superior performance of Random Forest in this study suggests that the additional hyperparameters and complexity of boosting methods did not provide an advantage over simpler Random Forest ensembles. The nonlinear effects and variable interactions present in our dataset appear well-suited for tree-based models, and the Random Forest algorithm effectively capitalized on these properties [50].

The performance metrics for the ML algorithms presented in this study align with recent research findings. The specific outcomes selected for this study have not been examined within a single study using ML algorithms before. However, several publications have explored the predictive performance of ML algorithms concerning postoperative outcomes following ACDF surgery using diverse data sources. For example, Gowd et al. employed ML models based on conventional comorbidity indices to compare predictive models for postoperative complications following ACDF surgery [21]. In this study, the logistic regression algorithm was the best performing for predicting any adverse event (AUROC = 0.73), transfusion (AUROC = 0.90), surgical site infection (AUROC = 0.63), and pneumonia (AUROC = 0.80), while gradient boosting trees was the best performing for predicting extended LOS (AUROC = 0.73). It is noteworthy that their study used ‘operative time’ as a predictor variable, and it was the most weighted variable for the prediction of any adverse event, extended LOS, and transfusion. Our study deliberately excluded variables like total operative time that would not be known prior to the surgery [51]. It must be kept in mind that instead of being the cause of undesirable outcomes, the length of the procedure might be a mediator [52]. Our study focuses on the preoperative prediction of adverse outcomes.

Rodrigues et al. queried the IBM MarketScan Commercial Claims and Encounters Database and Medicare Supplement from 2007 to 2016 to identify 176,816 patients who underwent an ACDF [22]. Some of the variables that were incorporated in the study were not available in the NSQIP database, such as operative characteristics, including bone morphogenic protein use, the use of anterior cervical plating, allograft or cage implants, and preoperative symptoms, including weakness, stiffness, or cervicalgia. Some of these variables for predicting 90-day readmissions, two-year reoperations, and 90-day complications were among the ones with high magnitudes of attention: myelopathy, human immunodeficiency virus (HIV), weakness, and stiffness. For the prediction of investigated outcomes, the deep neural network-based models in the study by Rodrigues et al. achieved AUROCs of between 0.671 and 0.832. Similarly, Khazanchi et al. investigated the predictive utility of ML and deep learning algorithms on postoperative health care utilization, including 90-day readmissions, postoperative LOS, and non-home discharge, in patients undergoing ACDF [25]. They utilized data from a multisite academic center and included a robust set of patient features, such as demographic information, medical/surgical history, operative characteristics, and preoperative lab values. The highest-performing model in their study was the Balanced Random Forest algorithm, with an AUROC of 0.70 for 90-day readmissions, 0.84 for non-home discharge, and 0.74 for extended LOS. Despite the reported performance and availability of granular data, these studies’ implications are largely exploratory due to the lack of an accessible tool for practical use.

Previously, Russo et al. proposed the novel ACDF Predictive Scoring System (APSS) algorithm using conventional statistics and ML to forecast LOS following one- or two-level ACDF surgery based on patient-specific preoperative characteristics and comorbidities [23]. The best-performing APSS model had an AUROC of 0.68. Although this study provides a form of tool to be used by clinicians, this study is limited by the low sample size of 1,506 patients and lower performance metrics. Arvind et al. also employed ML algorithms to predict complications following ACDF surgery using the NSQIP database [24]. Patients were excluded from the analysis in this study due to incomplete data, and no other exclusion criteria were employed. Although the case deletion method for handling the missing data is the most expedient method, it yields unbiased estimates only if the data are missing completely at random [53]. Likewise, not excluding emergency procedures, infections, tumor cases, trauma, and concomitant posterior approach surgeries increases the potential for preoperative confounding variables concerning surgical indications. In contrast, our model focuses specifically on predicting outcomes for degenerative cervical disease cases. Unfortunately, none of the aforementioned studies provided the source code for data preprocessing and classification models, limiting result reproducibility. Furthermore, none of these abovementioned studies offered a publicly accessible tool. In contrast, our web application provides interpretive predictions for three different outcomes, bridging the gap between complex ML predictions and their evaluation by healthcare professionals.

Our ML models and the associated web application provide individualized, quantitative estimates for unfavorable postoperative outcomes after ACDF. The presented approach represents a significant advancement over generalized risks derived from studies averaging across diverse populations, as well as the common practice of communicating risks qualitatively with some individual quantitative evaluation based on the clinician’s personal experience. However, relying solely on personal experience is constrained by inherently limited patient populations and potential subjective biases. The personalized predictions from our models can be used preoperatively to gauge prognosis during patient counseling, thus contributing to patient care. They allow healthcare professionals to identify patients at risk of certain adverse outcomes, prioritize their treatment, and plan for discharge requirements. Although the current web application provides a convenient interface for estimating the likelihood of adverse short-term postoperative outcomes following ACDF, it is intended as a research tool and should not currently guide clinical recommendations. Further validation in diverse patient cohorts across institutions is essential to confirm its predictive accuracy. We hope this calculator serves as a first step toward more comprehensive models that integrate additional factors like imaging findings and more granular clinical data for further refinement of predictive accuracy and clinical relevancy. As with any prediction tool, the estimates generated must be considered in the full context of each patient to personalize surgical counseling and planning.

Further limitations are similar to the limitations that have been described with other online prognostic models [52]. First, it is important to note that the patients in the ACS-NSQIP database may not be entirely representative of the general ACDF population. There may be biases related to the hospitals included in the database, as these hospitals may have above-average infrastructure and/or resources. Additionally, the patients in the database may have different health status, age, or socioeconomic backgrounds than the general population. Despite Huffman et al. demonstrating that the ACS-NSQIP database is a dependable data source for examining postsurgical outcomes and validating its usage, these limitations can affect the generalizability of our results [54]. Second, studies using a large clinical database are always influenced by coding errors and other inaccuracies. The NSQIP database is frequently used, but only a few studies have looked at it’s precision when it comes to coding. CPT codes for neurosurgical procedures contain numerous internal inconsistencies, according to Rolston et al. [55]. Furthermore, we did not compare our models’ performance to existing comorbidity indices or conduct external validation or user satisfaction analyses within the scope of the current study, which are important aspects to consider in future studies. Finally, we did not aim to identify causal relationships between patient characteristics and outcomes, and did not intend to suggest that our models could be used for causal inference or that they provide any information about the mechanisms underlying the observed associations between patient characteristics and outcomes. We do not encourage making causal interpretations based on the results of the current study.

In conclusion, our study has significantly improved the prediction of postoperative outcomes in patients undergoing ACDF surgery through the application of sophisticated ML methods. A key contribution of our work is the development of a user-friendly web application designed to provide a demonstration of the developed models’ practical utility. Our findings suggest that ML algorithms can serve as an invaluable auxiliary tool for patient risk stratification in ACDF surgery, with the potential to predict a variety of postoperative outcomes. This approach has the potential to play a critical role in counseling ACDF surgery patients, shifting the clinical approach towards a more patient-centric, data-driven model. Therefore, our study represents a substantial advancement in the field of precision medicine.

### Electronic supplementary material

Below is the link to the electronic supplementary material.

Supplementary Material 1

Supplementary Material 2

Supplementary Material 3

Supplementary Material 4

Supplementary Material 5

Supplementary Material 6

Supplementary Material 7

Supplementary Material 8

Supplementary Material 9

Supplementary Material 10

Supplementary Material 11

Supplementary Material 12

## Data Availability

Availability of Data and Materials: Data for this study were obtained from the American College of Surgeons (ACS) National Surgical Quality Improvement Program (NSQIP). ACS NSQIP participant use file access is a benefit of NSQIP participation and is reserved for staff at participating and active ACS NSQIP hospitals. ACS policies do not allow access or sale to non-participants. Additional information is provided in https://www.facs.org/quality-programs/data-and-registries/acs-nsqip/participant-use-data-file/.

## Declarations

### Statement by the ACS

The American College of Surgeons National Surgical Quality Improvement Program and the hospitals participating in the American College of Surgeons National Surgical Quality Improvement Program are the source of the data used herein; they have not verified and are not responsible for the statistical validity of the data analysis or the conclusions derived by the authors.

### Source code

The source code for preprocessing and analyzing the data is available on GitHub (https://github.com/mertkarabacak/NSQIP-ACDF).

## Abbreviations

ACDF: Anterior cervical discectomy and fusion
APSS: ACDF Predictive Scoring System
ASA: American Society of Anesthesiologists
AUROC: The area under the receiver operating characteristic
AUPRC: Area under the precision-recall curve
BUN: Blood urea nitrogen
COPD: Chronic obstructive pulmonary disease
CPT: Current Procedural Terminology
HIV: Human immunodeficiency virus
ICD: International Classification of Diseases
JMIR: Journal of Medical Internet Research
LOS: Length of stay
ML: Machine learning
NSQIP: National Surgical Quality Improvement Program
PTT: Partial thromboplastin time
PT: Prothrombin time
PRC: Precision-recall curve
ROC: Receiver operating characteristic
SGOT: Serum glutamic-oxaloacetic transaminase
SHAP: SHapley Additive exPlanations
SMOTE: Synthetic Minority Over-sampling Technique
TPESampler: Tree-Structured Parzen Estimator Sampler
TRIPOD: Transparent Reporting of Multivariable Prediction Models for Individual Prognosis or Diagnosis
WBC: White blood cell
