# Supplementary material for: Machine learning models on a web application to predict short-term postoperative outcomes following anterior cervical discectomy and fusion
Source: BMC Musculoskelet Disord. 2024 May 21;25:401. doi: 10.1186/s12891-024-07528-5 (PMC11110429; doi:10.1186/s12891-024-07528-5)
Supplement: Supplementary file 1 — Supplementary Material 1 [file 12891_2024_7528_MOESM1_ESM.docx]

**Supplementary Figure 1.** The 15 most important features and their mean SHAP values for the models predicting the outcome prolonged length of stay with the A) TabPFN, B) TabNet, C) XGBoost, and D) LightGBM algorithms.

**
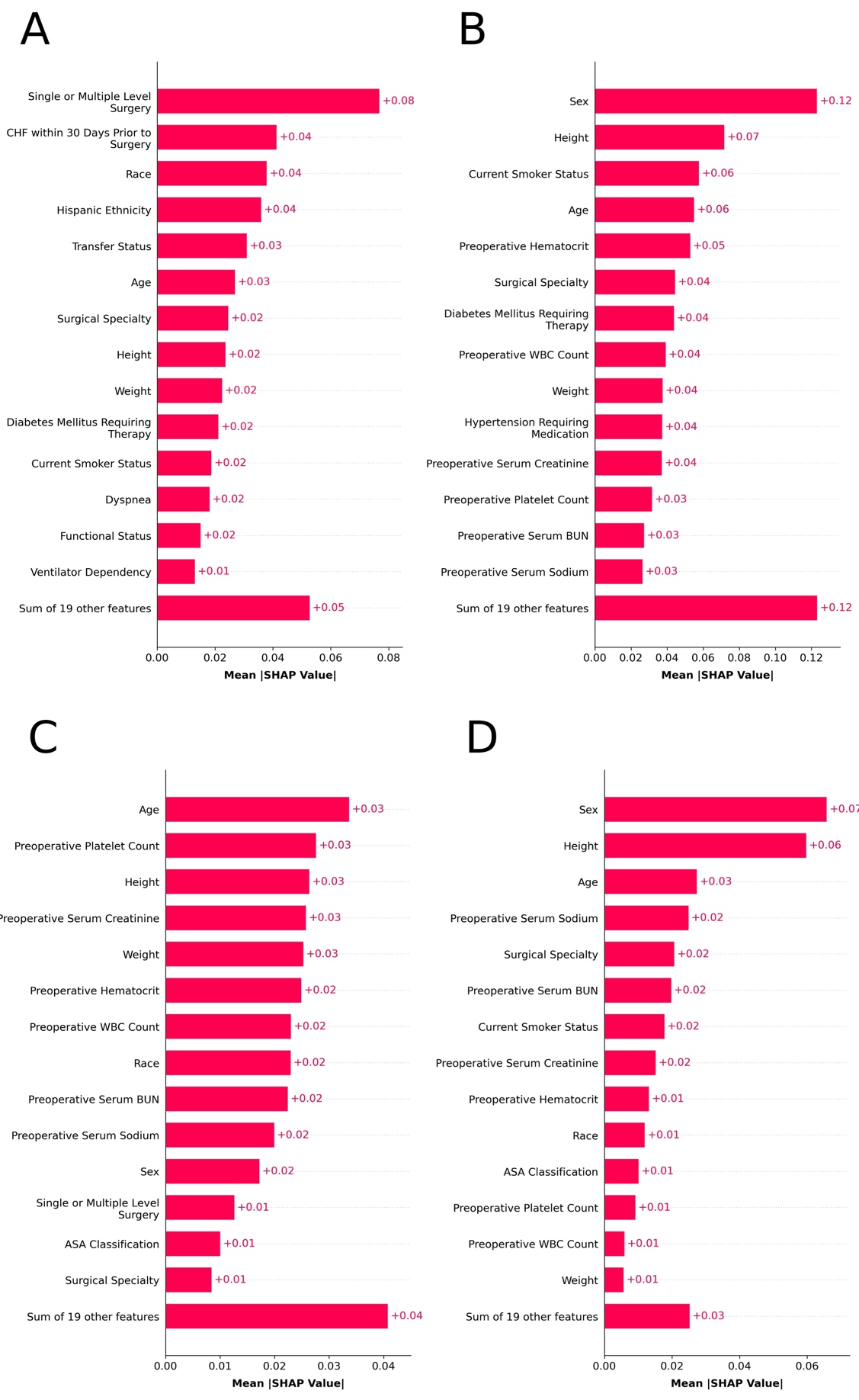
**
