# Supplementary material for: Machine learning models on a web application to predict short-term postoperative outcomes following anterior cervical discectomy and fusion
Source: BMC Musculoskelet Disord. 2024 May 21;25:401. doi: 10.1186/s12891-024-07528-5 (PMC11110429; doi:10.1186/s12891-024-07528-5)
Supplement: Supplementary file 2 — Supplementary Material 2 [file 12891_2024_7528_MOESM2_ESM.docx]

**Supplementary Figure 2.** The 15 most important features and their mean SHAP values for the models predicting the outcome non-home discharges with the A) TabPFN, B) TabNet, C) XGBoost, and D) LightGBM algorithms.

**
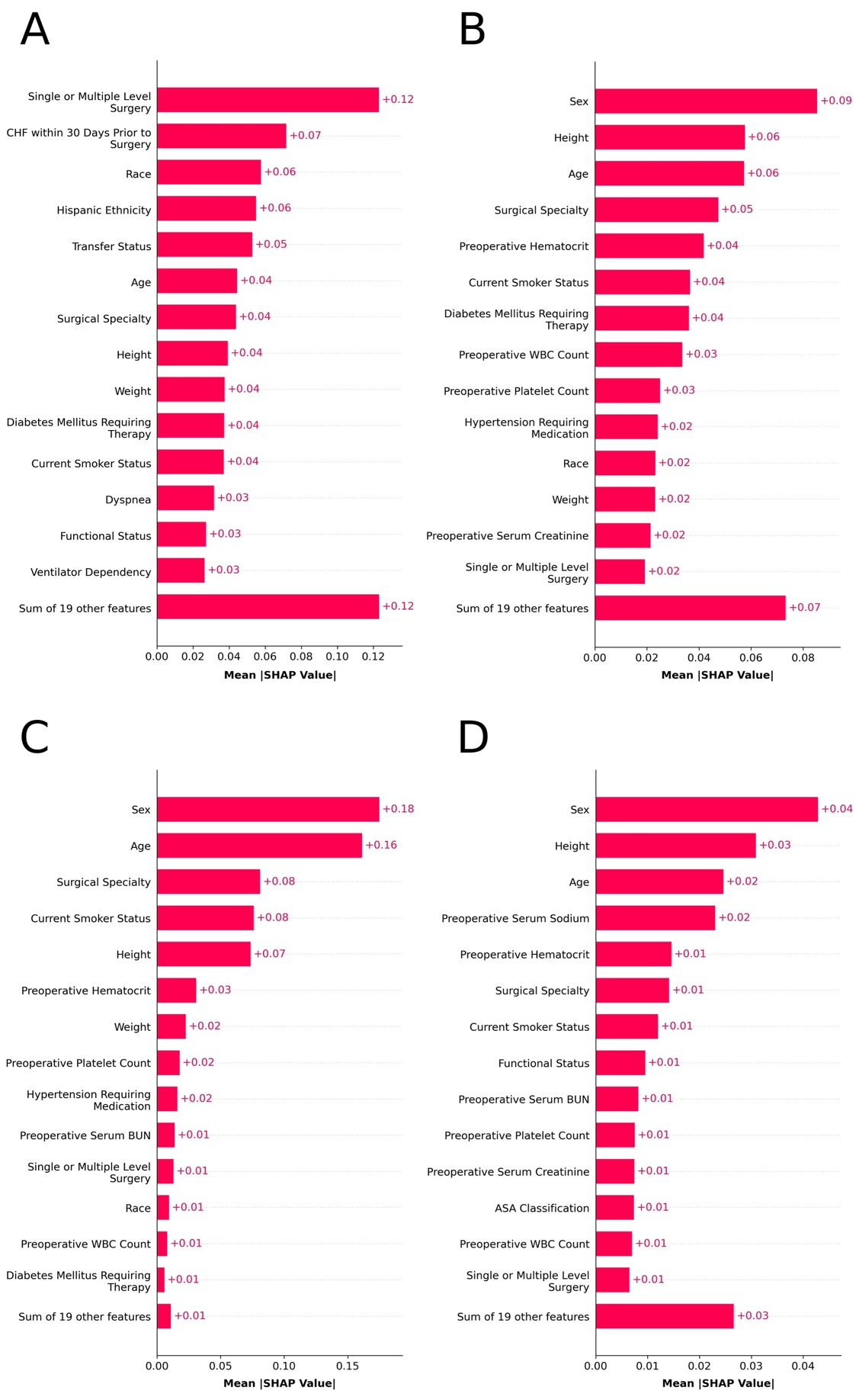
**
