# Supplementary material for: Machine learning models on a web application to predict short-term postoperative outcomes following anterior cervical discectomy and fusion
Source: BMC Musculoskelet Disord. 2024 May 21;25:401. doi: 10.1186/s12891-024-07528-5 (PMC11110429; doi:10.1186/s12891-024-07528-5)
Supplement: Supplementary file 6 — Supplementary Material 6 [file 12891_2024_7528_MOESM6_ESM.docx]

**Supplementary Figure 6.** The partial dependence plot for the 9 most important features of the Random Forest model predicting non-home discharges showing the global influence of individual variables on the predictions.

**
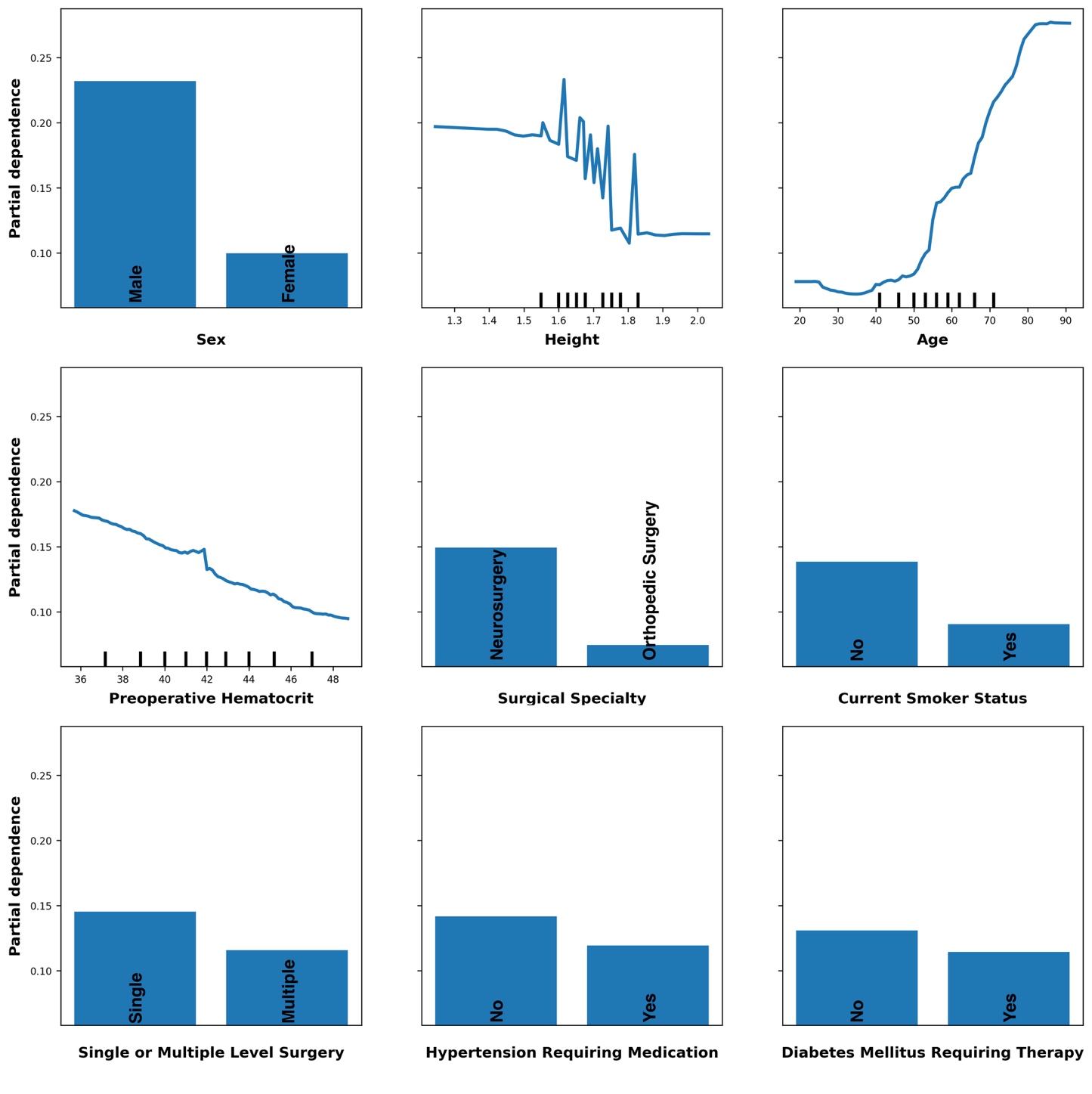
**
