# Supplementary material for: Machine learning models on a web application to predict short-term postoperative outcomes following anterior cervical discectomy and fusion
Source: BMC Musculoskelet Disord. 2024 May 21;25:401. doi: 10.1186/s12891-024-07528-5 (PMC11110429; doi:10.1186/s12891-024-07528-5)
Supplement: Supplementary file 9 — Supplementary Material 9 [file 12891_2024_7528_MOESM9_ESM.docx]

**Supplementary Table 1:** CPT codes used for inclusion.

| **CPT** | **CPT description** |
| --- | --- |
| 22551 | Arthrodesis, anterior interbody, including disc space preparation, discectomy, osteophytectomy and decompression of spinal cord and/or nerve roots; cervical below C2 |
| 22552 | Arthrodesis, anterior interbody, including disc space preparation, discectomy, osteophytectomy and decompression of spinal cord and/or nerve roots; cervical below C2, each additional interspace |
| 22554 | Arthrodesis, anterior interbody technique, including minimal discectomy to prepare interspace (other than for decompression) |
| 22585 | Arthrodesis, anterior interbody technique, including minimal discectomy to prepare interspace; each additional interspace |
