# Supplementary material for: Machine learning models on a web application to predict short-term postoperative outcomes following anterior cervical discectomy and fusion
Source: BMC Musculoskelet Disord. 2024 May 21;25:401. doi: 10.1186/s12891-024-07528-5 (PMC11110429; doi:10.1186/s12891-024-07528-5)
Supplement: Supplementary file 10 — Supplementary Material 10 [file 12891_2024_7528_MOESM10_ESM.docx]

**Supplementary Table 2:** CPT codes used for exclusion.

| **CPT** | **CPT description** |
| --- | --- |
| 22590 | Posterior, Posterolateral or Lateral Transverse Process Technique Arthrodesis Procedures on the Spine (Vertebral Column) |
| 22595 | Arthrodesis, posterior technique, atlas-axis (C1-C2) |
| 22600 | Arthrodesis, posterior or posterolateral technique, single level; cervical below C2 segment |
| 22614 | Each additional vertebral segment (list separately in addition to code for primary procedure) |
| 22856 | Total disc arthroplasty (artificial disc) anterior approach, including discectomy to prepare  interspace (other than for decompression) single interspace: cervical |
| 22858 | Total disc arthroplasty (artificial disc), anterior approach, including discectomy with end plate  preparation (includes osteophytectomy for nerve root or spinal cord decompression and  microdissection), second level, cervical (list separately in addition to code for primary procedure) |
| 22861 | Revision including replacement of total disc arthroplasty (artificial disc), anterior approach, single interspace; cervical |
| 22864 | Removal of total disc arthroplasty (artificial disc), anterior approach, single interspace; cervical |
