# Supplementary material for: Machine learning models on a web application to predict short-term postoperative outcomes following anterior cervical discectomy and fusion
Source: BMC Musculoskelet Disord. 2024 May 21;25:401. doi: 10.1186/s12891-024-07528-5 (PMC11110429; doi:10.1186/s12891-024-07528-5)
Supplement: Supplementary file 11 — Supplementary Material 11 [file 12891_2024_7528_MOESM11_ESM.docx]

**Supplementary Table 3:** Model hyperparameters.

| **Outcome** | **Algorithm** | **Hyperparameters** |
| --- | --- | --- |
| Prolonged Length of Stay | TabPFN | None. |
|  | TabNet | {'n_d': 12, 'n_a': 38, 'n_steps': 1, 'gamma': 1.3823932675263133, 'n_independent': 4, 'n_shared': 3, 'lambda_sparse': 1.354780454607063e-06} |
|  | XGBoost | {'objective': 'binary:logistic', 'booster': 'gbtree', 'lambda': 8.933240120218292e-08, 'alpha': 2.465186460458053e-07, 'max_depth': 9, 'eta': 0.0016622545171405692, 'gamma': 0.0038171544493266004, 'grow_policy': 'lossguide', 'eval_metric': 'auc', 'verbosity': 0, 'seed': 31} |
|  | LightGBM | {'objective': 'binary', 'boosting_type': 'gbdt', 'lambda_l1': 2.9361485404205944e-05, 'lambda_l2': 0.054104326505040545, 'num_leaves': 256, 'feature_fraction': 0.8987167941544422, 'bagging_fraction': 0.8313988120314314, 'bagging_freq': 3, 'min_child_samples': 7, 'metric': 'binary_logloss', 'verbosity': -1, 'random_state': 31} |
|  | Random Forest | {'criterion': 'gini', 'max_features': 'sqrt', 'max_depth': 53, 'n_estimators': 200, 'min_samples_leaf': 1, 'min_samples_split': 2, 'random_state': 31} |
| Non-home Discharges | TabPFN | None. |
|  | TabNet | {'n_d': 24, 'n_a': 62, 'n_steps': 8, 'gamma': 1.9868700030924629, 'n_independent': 2, 'n_shared': 1, 'lambda_sparse': 0.0005310328735364063} |
|  | XGBoost | {'objective': 'binary:logistic', 'booster': 'gbtree', 'lambda': 0.01268558739073288, 'alpha': 0.00040620213261866983, 'max_depth': 8, 'eta': 0.08773119436773698, 'gamma': 0.04703455580243, 'grow_policy': 'depthwise', 'eval_metric': 'auc', 'verbosity': 0, 'seed': 31} |
|  | LightGBM | {'objective': 'binary', 'boosting_type': 'gbdt', 'lambda_l1': 0.001255294207701933, 'lambda_l2': 0.0001634010228214476, 'num_leaves': 253, 'feature_fraction': 0.9729632286118172, 'bagging_fraction': 0.9445732924730252, 'bagging_freq': 1, 'min_child_samples': 26, 'metric': 'binary_logloss', 'verbosity': -1, 'random_state': 31} |
|  | Random Forest | {'criterion': 'gini', 'max_features': 'sqrt', 'max_depth': 53, 'n_estimators': 200, 'min_samples_leaf': 1, 'min_samples_split': 2, 'random_state': 31} |
| 30-Day Readmissions | TabPFN | None. |
|  | TabNet | {'n_d': 36, 'n_a': 23, 'n_steps': 4, 'gamma': 1.9744718514900728, 'n_independent': 1, 'n_shared': 2, 'lambda_sparse': 0.00011786312212022038} |
|  | XGBoost | {'objective': 'binary:logistic', 'booster': 'gbtree', 'lambda': 8.104693554106028e-05, 'alpha': 1.1096183177943485e-05, 'max_depth': 9, 'eta': 0.00313617849552747, 'gamma': 0.26625409466396327, 'grow_policy': 'depthwise', 'eval_metric': 'auc', 'verbosity': 0, 'seed': 31} |
|  | LightGBM | {'objective': 'binary', 'boosting_type': 'gbdt', 'lambda_l1': 0.0005779879446540671, 'lambda_l2': 3.1155196998363778e-06, 'num_leaves': 252, 'feature_fraction': 0.8103994108194666, 'bagging_fraction': 0.9896530930870733, 'bagging_freq': 2, 'min_child_samples': 24, 'metric': 'binary_logloss', 'verbosity': -1, 'random_state': 31} |
|  | Random Forest | {'criterion': 'gini', 'max_features': 'sqrt', 'max_depth': 53, 'n_estimators': 200, 'min_samples_leaf': 1, 'min_samples_split': 2, 'random_state': 31} |
| Major Complications | TabPFN | None. |
|  | TabNet | {'n_d': 36, 'n_a': 23, 'n_steps': 4, 'gamma': 1.9744718514900728, 'n_independent': 1, 'n_shared': 2, 'lambda_sparse': 0.00011786312212022038} |
|  | XGBoost | {'objective': 'binary:logistic', 'booster': 'gbtree', 'lambda': 0.13842433262171935, 'alpha': 0.004889527666207119, 'max_depth': 2, 'eta': 0.1265633609710301, 'gamma': 0.014013601715970112, 'grow_policy': 'lossguide', 'eval_metric': 'auc', 'verbosity': 0, 'seed': 31} |
|  | LightGBM | {'objective': 'binary', 'boosting_type': 'gbdt', 'lambda_l1': 4.866915220362586e-06, 'lambda_l2': 1.5287766879596804e-08, 'num_leaves': 249, 'feature_fraction': 0.45983596850715286, 'bagging_fraction': 0.7865081197362238, 'bagging_freq': 4, 'min_child_samples': 16, 'metric': 'binary_logloss', 'verbosity': -1, 'random_state': 31} |
|  | Random Forest | {'criterion': 'gini', 'max_features': 'sqrt', 'max_depth': 53, 'n_estimators': 200, 'min_samples_leaf': 1, 'min_samples_split': 2, 'random_state': 31} |
